# Redesigning the Process for Scheduling Elective Orthopaedic Surgery: A Combined Lean Six Sigma and Person-Centred Approach

**DOI:** 10.3390/ijerph182211946

**Published:** 2021-11-13

**Authors:** Ailish Daly, Nicola Wolfe, Seán Paul Teeling, Marie Ward, Martin McNamara

**Affiliations:** 1 Beacon Hospital Beacon Court, Bracken Rd, Sandyford Business Park, Sandyford, D18 AK68 Dublin, Ireland; Nicola.wolfe@beaconhospital.ie; 2 UCD Centre for Interdisciplinary Research, Education & Innovation in Health Systems, School of Nursing, Midwifery & Health Systems UCD Health Sciences Centre, D04 V1W8 Dublin, Ireland; sean.p.teeling@ucd.ie (S.P.T.); martin.mcnamara@ucd.ie (M.M.); 3 Centre for Person-Centered Practice Research Division of Nursing, School of Health Sciences, Queen Margaret University Drive, Musselburgh EH21 6UU, East Lothian, UK; 4 Centre for Innovative Human Systems, School of Psychology, Trinity College, The University of Dublin, D02 PN40 Dublin, Ireland; marie.Ward@tcd.ie

**Keywords:** elective surgery, scheduling, Lean Six Sigma, collaborative, voice of customer, cross-functional team

## Abstract

The Health Service Executive Ireland model of care for elective surgery supports the delivery of elective surgical care in achieving both process and clinical outcomes. This project was conducted in the Orthopaedic Department. Following an outpatient consultation with an orthopaedic surgeon, patients who required surgical intervention were scheduled for their intervention by the administrative team. Prior to commencing this project, the average time from patient consultation to being scheduled for surgery on the hospital system was 62 h/2.58 days. A pre- and post-team-based intervention design employing Lean Six Sigma methodology was applied to redesign the process for scheduling elective orthopaedic surgery. The project was informed by collaborative, inclusive, and participatory stakeholder engagement. The goal was to streamline the scheduling process for elective orthopaedic surgery, with a target that 90% of surgeries are scheduled “right first time” within 48 h/two working days of the outpatient consultant appointment. The main outcome measures showed that 100% of orthopaedic surgeries were scheduled successfully within 2 days of outpatient appointment. Duplication in work between patient services and scheduling teams was eliminated and facilitated a reduction in unnecessary staff workload. This project highlights the importance of collaborative interdisciplinary stakeholder engagement in the redesigning of processes to achieve sustainable outcomes, and the findings have informed further improvements across the hospital’s surgical scheduling system.

## 1. Introduction

The setting for this project is a large private hospital in Dublin. The hospital has a strong improvement culture, with 250 staff trained in Lean Six Sigma (LSS) methodology by our academic partner University College Dublin. The university program has been instrumental in the development of LSS healthcare education and training nationally [1]. As part of its ongoing LSS improvement work, in 2018 the hospital undertook a review of the process of patient scheduling using the orthopaedic surgery service for an improvement intervention.

Lean Six Sigma is a powerful methodology that reduces waste and variation in an organisation, and ultimately minimises operating costs, optimises productivity, and maximises customer satisfaction [2]. LSS is the merger of two methods used in process improvements. Lean originated in Toyota car production factories, and focuses on refining and improving processes as well as eliminating non-value-added (NVA) activities [3] Six Sigma was introduced by Motorola to optimise its manufacturing processes by reducing their variability through the rigorous application of process metrics collection and statistical analysis [4,5] Since the early 2000s, LSS thinking has been adapted into healthcare with the goal of improving patient safety, quality of care, efficiency, patient satisfaction, and performance [6].

As healthcare providers worldwide, both publicly and privately funded, are faced with similar challenges of caring for an ageing population with a limited pool of financial and personnel resources, the need to seek improved efficiencies while continuing to provide quality services has become more and more acute [7]. LSS has been implemented in many healthcare organisations, with impacts achieved across many clinical and administrative pathways and processes [8,9] Especially in orthopaedics, LSS has been utilised to reduce the length of stays for patients undergoing knee replacement [10,11]

A surgical patient’s successful journey from initial consultation to actual surgical intervention relies on a complex group of clinical and administrative processes. Clinically, the work of a surgeon and a wider multidisciplinary team (MDT) ensures the completion of appropriate assessments and pre-operative clinical preparation to enable patients to be admitted to hospitals for elective surgery [12]

Supporting this clinical preparation is a complex set of administrative processes, crossing multiple departments within the organisation, that ensure that correct patient information is available to all members of the clinical and support teams at every point of a patient’s journey. Failure to schedule patients correctly following consultation has a wide variety of potential system-wide consequences, ranging from cancellations or delays in the operating room (OR) to the administrative team duplicating process steps [13] 

Patients are referred for surgery by surgeons who attend outpatient clinics, both internal and external to the hospital. To ensure timely elective surgery, the hospital requires the submission of a scheduling form (called the booking form in the project site) no less than 5 days pre-surgery. Urgent cases are accepted depending on a patient’s acuity. The scope of this project includes elective orthopaedic cases (pre-planned surgeries), but not cases performed in an emergency situation. Elective orthopaedic cases, according to the World Health Organisation, are related to musculoskeletal conditions that are typically characterised by persistent pain and restricted mobility [14] 

The most common and disabling musculoskeletal conditions are back and neck pain as well as systemic inflammatory conditions, such as rheumatoid arthritis. Timely access to orthopaedic surgery is essential for optimal patient outcomes and ensures that patients can return to most of their normal activities [13] Simon and Canceri describe the process and potential challenges in scheduling elective orthopaedic surgery. They mention challenges associated with a paper-based system, time lag between confirming with the patient that surgery is required and confirming the date of surgery, inaccurate booking information, last-minute changes, and workarounds due to inaccurate information.

Within the project site, 300 elective orthopaedic surgeries are performed monthly by up to 16 different surgeons from outpatient clinics that are both internal, within the campus of the hospital, and external to the project site hospital. To facilitate this variation in the offsite versus onsite booking forms the hospital operated a hybrid scheduling system that facilitated both hardcopy and online booking forms. Sixteen different versions of the hardcopy booking form had been developed and were in circulation following previous attempts at improving the process in 2016. It was noted by those working with the scheduling process (process users) that the content of the hardcopy booking form did not match the content of the online scheduling system. An initial request for feedback from staff who were the process users (*n* = 10) indicated that they found the scheduling process to be “time-consuming”, booking forms were often incomplete with reworks (carrying out the same process steps again) required. Initial patient feedback (*n* = 10) indicated frustration that the date of the surgery was not always confirmed at their outpatient clinic appointment, as well as frustration at what they felt was repeated questioning regarding past medical history and demographics.

**Figure 1:**
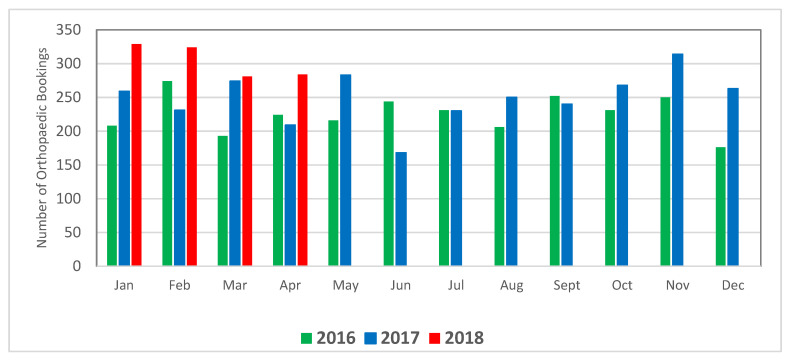
Number of orthopaedic surgeries scheduled for 2016, 2017, and 2018.

In the years from 2015 to 2018, the hospital experienced a year-on-year increase in demand for their scheduled orthopaedic surgery service and a corresponding increase in operating room (OR) activity (Figure 1). There was also an increase in patient acuity and case complexity, for example, in the introduction of robotic procedures to the hospital. This increased demand for more complex elective surgeries meant that the correct completion of elective surgery booking forms, more than ever, needed to be right first time to avoid any delay in scheduling and to maximise the utilisation of available OR slots. As all surgeries within the hospital across all 10 specialties used the same scheduling system, the hospital management team felt that any improvement in orthopaedic elective surgery scheduling would have an impact on the entire scheduling system. Therefore, a project team was convened and supported by the management team with the intention of improving the existing scheduling process. This use of cross-functional LSS teams working outside their practice areas enables the organisation to overcome professional and departmental silos that can act as barriers to the successful implementation of system-wide process improvements [8,9] 

## 2. Methods

A pre- and post-team-based intervention design was employed by utilising the LSS define/measure/analyse/improve/control (DMAIC) framework to structure this project [15].

A project goal was agreed with support from the hospital CEO/Deputy CEO with a defined project target of 100% of elective orthopaedic surgeries being scheduled correctly, the first time, on the hospital’s electronic record system, MEDITECH, within 2 days of an outpatient appointment. This goal would be achieved through defining and agreeing on a minimum data set (MDS) for the hardcopy booking form and reducing NVA as well as rework in the scheduling process. NVA refers to any work activity that consumes resources, but does not add value or contribute to the “customer”—in the context of healthcare, this can be staff activity or the patient’s care [5]. The project scope included only scheduling for elective procedures; no trauma or emergency case surgeries were included.

Utilising LSS tools enables improvement teams to spot and rectify bottlenecks in processes to facilitate process improvement. They also provide a comprehensive set of tools to facilitate engagement with customers involved in the process to enable meaningful change, rapid root cause analysis, and genuine staff involvement [4] with staff leading on projects to improve patient outcomes [16,17]. Table 1 outlines the LSS tools that were utilised in the process improvement outlined in this project, and the rationale for the tools use.

### 2.1. Define Phase

At a high-level view, the process to schedule a patient for surgery had four key steps, which began with the first step of the surgeon handwriting the patient and surgery details onto a hardcopy booking form. This was then handed to the patient services team to:

(a). Send to the scheduling team to enter it into the hospital information system, MEDITECH (hardcopy).

**Figure 2:**
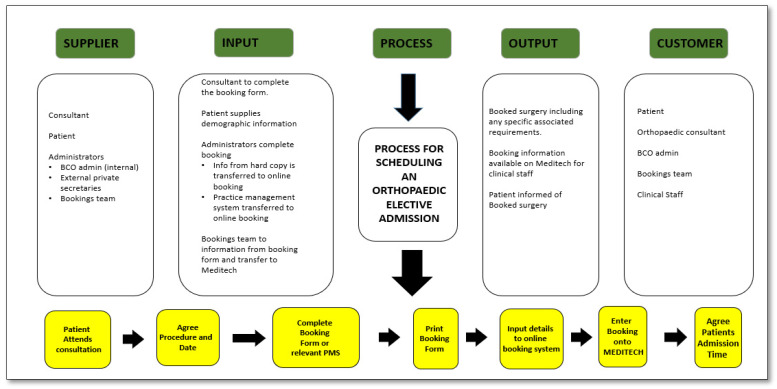
SIPOC.

Finally, as a fourth step, the patient’s surgery was then confirmed to the patient by text message. This process was made visible by the use of an LSS SIPOC tool (Figure 2), which facilitates a high-level view of the process [4].

Once our goal was agreed a communication plan was formulated to facilitate stakeholder engagement. The communication plan was used to ensure we built a commitment to the project by talking to the right people at every stage. This allowed our stakeholders to be more engaged and confident, as they understood what we were doing, why we were doing it, and how the project was progressing [5]. 

Key participating staff were identified as the orthopaedic patient services team, orthopaedic consultants, and the OR scheduling team, with the full support of the executive management team (EMT), including the CEO and the Head of Patient Services. Cognisant of the role of staff in this improvement, we held multiple voice of the customer (VOC) sessions designed to capture our customers’ expectations of the improvement and their initial thoughts on the current process [20] VOC sessions were conducted through focus groups or individual interviews. Participants were selected by purposive sampling of the outlined key participating staff. We used a purposive sample design to enable the generation of data on the scheduling process, draw clear inferences and credible explanations from the data that were generated, and be as efficient as was practical [24,25]

Focus groups allowed for the sharing of ideas and seeing challenges from the perspective of others, whereas individual interviews allowed for every team member, regardless of seniority or position, to have a forum to contribute their ideas. The VOC has also been shown to be synergistic with person-centred approaches to improvement [26]. Specific feedback from key participating staff included:

Team member 1: “can take anything from 5 min to 5 days to schedule!”;
Team member 2: “Too many clicks and pages to navigate online scheduling”;
Team member 3: “Insurance details are often missing”;
Team member 4:” Unclear handwriting on paper form makes entering online scheduling difficult”;
Team member 5: “Booking form is in landscape and all other documents are in portrait form in the Medical record”;
Team member 6: “Procedure codes and descriptions don’t match”.

**Figure 3:**
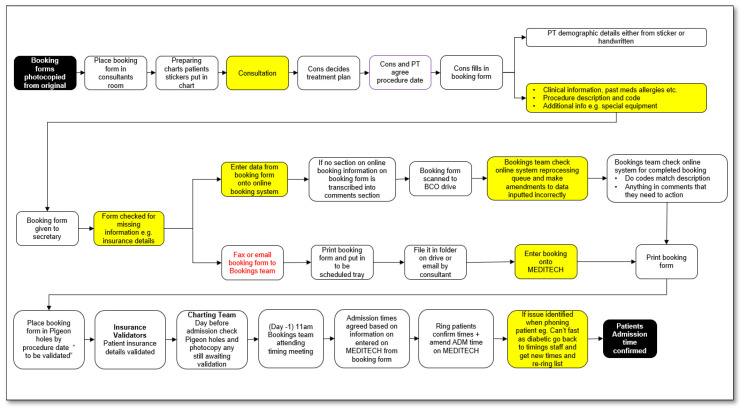
“As it is processed” map.

During the initial VOC sessions, we developed an understanding of what we thought the process, which seemed straightforward and involved six steps to schedule a patient for elective orthopaedic surgery, reflected in the developed SIPOC (Figure 2). However, subsequent VOC sessions held to discuss the process and collaboratively develop a more detailed process map with participating staff resulted in a far more complex process, comprising 24 touchpoints (Figure 3). The outputs from our VOC sessions were sorted using thematic analysis of workshop and interview outputs, a common analysis technique for qualitative research [27]. Key themes that emerged from the VOC were challenges in completing the process when not all the required information was included in the booking form, time taken, repeated work in completing online booking, as well as the challenge in reading illegible handwriting.

### 2.2. Measure Phase

A more detailed process map was completed to show “as it is processed” (Figure 3). This facilitated the identification and classification of the types of waste, and we utilised the LSS acronym TIMWOODS (transportation, inventory, movement, waiting, overprocessing, overproduction, defects, and skills) [23]. The waste in the process is illustrated in Figure 3 (denoted in yellow), and illustrated areas where it was possible to collect data to relate to time, accuracy, and where work was not completed accurately the first time (rework). This process map also illustrated that the 6-step process outlined at a high level in the SIPOC (Figure 2) actually contains 24 touchpoints as outlined in Figure 3—“as it is processed” map.

The leap to 24 touchpoints is reflective of a busy healthcare environment, where busy hospital staff work in departmental silos and do not see the entire service [28,29,30]. Additionally, in healthcare, as in other industries, some workflows are designed while others evolve or develop organically [31] 

VOC sessions also facilitated the development of a critical to quality tree (CTQ), a tool that is used in LSS to translate the voice of a customer into quantifiable metrics [18]. Key metrics identified for the collection were:

1. Surgeries booked: the percentage of surgeries scheduled on the online scheduling system successfully at first attempt (both hardcopy and online methods).
2. Time taken: time taken from being seen in the Outpatient Department (OPD) to patient scheduled for surgery.
3. OR schedule: any corresponding changes to OR schedule.

Data collection was completed by Gemba [21] (Japanese term for going to and observing the process where value is created) and an audit over a period of 8 weeks, through observing the process and identifying each interaction or “touchpoints” of the booking form from the consultant’s room through to the completion on the scheduling system. An audit was completed through data mining of the hospital electronic patient record/MEDITECH to confirm the time of surgery scheduling completion.

**Figure 4:**
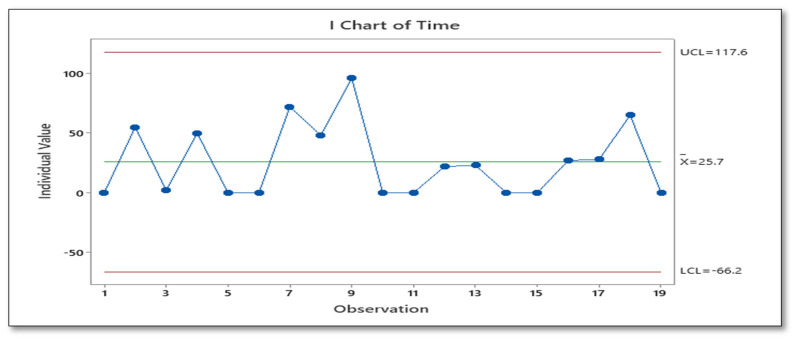
Time from OPD appointment to the scheduling of surgery.

Table 2 and Figure 4 shows that time taken to complete an online booking ranged from immediate to 96 h with a mean of 28 h. Immediate online booking was achieved when all required information was available and correct on the booking form, therefore surgery could be scheduled successfully (Table 3). When information was not available or unclear online booking could not be completed immediately. It could take up to 96 h from when an online booking was commenced to and clarify and correct information required to complete an online booking.

### 2.3. Analyse Phase

**Figure 5:**
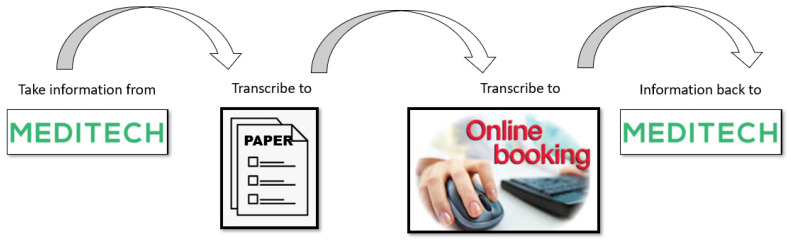
Duplication of work.

Analysis revealed multiple findings detailed below:

- Patient services staff accessed up to 9 different IT platforms (Figure 5) to obtain data/missing information required to schedule a patient for surgery. Platforms include internal systems such as MEDITECH, an online booking system, a ShoreTel (phone) system, consultant dictation systems (IMEDOC), external systems such as Google, and insurance systems including Claimsure, VHI, Laya, and Irish Life validation systems.
- Data that originated in the hospital electronic patient record (EPR) were transferred to a paper format, then to a hospital online scheduling system and finally back to MEDITECH (Figure 4).
- Completion of the online scheduling system, based on an average of 5 Gembas performed over a 1-week period, required 105 fields which the team were unable to bypass, therefore utilising 105 clicks of a computer mouse.
- There was no categorisation or SMART (specific, measurable, achievable, realistic/relevant, and timed) grouping of procedure code within online scheduling—for example, total knee replacement always takes place in the OR and is always an overnight stay. However, administration staff have to click OR as the location and an overnight stay each time a total knee replacement is scheduled.
- Potential opportunities for error existed, for example, there were 5 different procedure descriptions assigned to the same procedure code.
- It took on average 4 min to complete the paper booking form (*n* = 10) by 3 different surgeons.
- There were 19 online booking forms scheduled over a period of 3 days (N = 19):
  ○ Minimum value = 2 min, no rework required.
  ○ Median value = 62 h, rework required.

Overall, the administrative process to schedule an elective orthopaedic surgery could take 62 h, and the surgery itself could take 30 min in the case of an injection to 2.5 h for joint replacement surgery.

The project team completed a failure mode effect analysis (Table 4) to evaluate the process, identify where and how it might fail, and assess the relative impact of different failures [22]. The potential severity, occurrence, and detection of potential failures were scored using a 0–10 scale, with 10 indicating high severity, high likelihood of occurrence, and unlikely to be detected. This involves developing risk prioritisation numbers (RPN) which is a numeric assessment of the risk assigned to a process. This highlighted the main risk areas as the completion of online scheduling, followed by the completion of the booking form by orthopaedic consultants, with RPN scores of 420 and 400, respectively.

### 2.4. Improve Phase

We presented the results of our Gemba and our data collection to our stakeholders and facilitated collaborative, inclusive, and participative brainstorming sessions (*n* = 3) to identify potential improvements [20]. Suggestions for improvements were collaboratively designed by project participants and broadly fell into three key improvement streams:

1. Process redesign and how to improve the quality of information we give and receive.
2. Expertise and how to optimise staff skills and their valuable time.
3. Stakeholder engagement and continuous education.

We expand on the co-designed solutions below.

#### 2.4.1. Improvement 1: Redesign and Improve the Quality of Information We Give and Receive

A specific Lean tool, “5 S” (visual workplace management), is used to create a work environment that is clean, well-organised, and efficient [6,18]. An adapted version of the Lean 5 S principles (Table 5) was applied to the paper booking form to focus on an agreed minimum data set and a reduction in the number of fields.

Quick wins included updating:

- Open-text medical history fields to specific yes/no fields, identifying the presence or absence of pre-operative conditions that may increase a patient’s risk of complications during surgery;
- Elimination of fields that were found by stakeholders and process owners as not actually being required;
- Using the space gained to allow for increased font size and improved readability;
- Agreed change from a portrait to a landscape view, consistent with all other forms in the hospital medical record.

Additional functionality was built into the online scheduling system, such as key repeatable features aligned to each surgery—for example, total hip replacement is always completed in the OR, so this location is now an autofill function—rather than the consultant having to choose a location from a list including the OR, endoscopy, and cardiology procedure suites (Table 6).

#### 2.4.2. Improvement 2: Experts and Optimising Staff Resources

Participants agreed that orthopaedic patient services staff would use their expertise and knowledge of the process to carry out a daily audit on scheduling forms for completion, and that the specialist scheduling team would schedule on the hospital scheduling system (MEDITECH). In doing so, the removal the duplication of work identified in the analysis phase of DMAIC between patient services and the scheduling team was achieved.

#### 2.4.3. Improvement 3: Engagement and Compliance

Our collaborative, inclusive, and participatory stakeholder meetings led to the development of a co-designed team approach to the process improvement. It was agreed that they would be the catalyst of a new user group to monitor the improvement in the orthopaedic scheduling process, and the meetings led to a new series of monthly, regular (specify weekly, monthly, etc.) follow-up engagement sessions with main process users/stakeholders, including consultants, consultants’ secretaries, and patient services staff.

**Figure 6:**
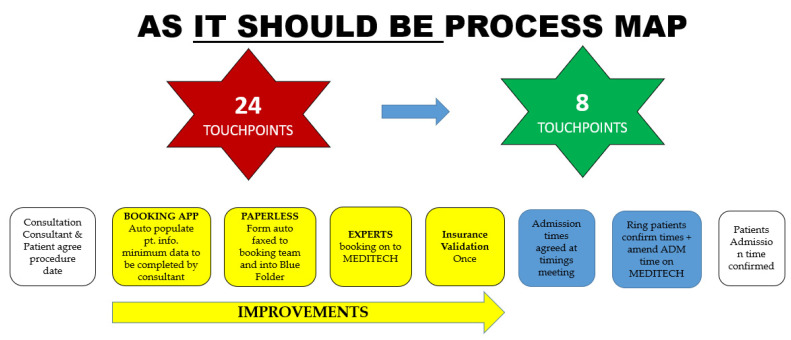
As it should be process.

Following the implementation of the discussed improvements, the process map was updated (Figure 6) to illustrate the impact of the improvements. The impact of the solutions is reflected in our results.

## 3. Results

**Figure 7:**
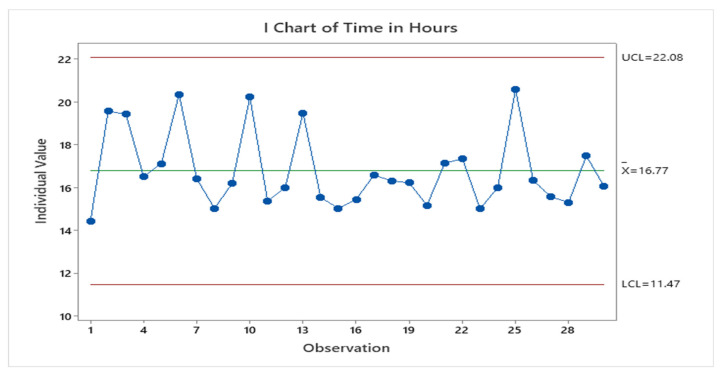
Time from OPD appointment to the scheduling of surgery.

On completion of this project, an audit of elective orthopaedic surgeries scheduled (*n* = 30) by three different consultants over a 3-week period indicated that they were completed within two working days of outpatient appointments (Table 7 and Figure 7). This effectively meant that patients were scheduled for surgery sooner—allowing patients to focus on preparing for their surgery. This was repeated after a control period of 6 months, and compliance remained with bookings scheduled within two working days of outpatient appointment.

### Descriptive Statistics

Per annum, 92 h of patient services time was saved. There was a 50% reduction in the time taken to complete the newly formatted booking form, releasing consultants’ and patient services staffs’ time to engage with their patients, answering questions regarding their upcoming surgery rather than spending time completing a form. Feedback from the VOC on the improved process included feedback on the LSS approach as well as feedback on the new scheduling process:

- Team member one: “Instead of coming in and telling us what we were doing wrong we were listened to”—this reflects the person-centred approach to LSS process improvement [26];
- Team member two: “We can already start to see improvements”;
- Team member three: “The new form is so much easier to read”;
- Team member four: “We did not realise there was so much information available on MEDITECH”.

## 4. Discussion

Through utilising an LSS approach, the project team succeeded in achieving the completion of 100% of scheduled, elective orthopaedic surgeries within two working days of an outpatient appointment. This success was achieved through:

1. Collaborative working to redesign the new booking form and scheduling process;
2. Facilitating the stakeholder experts in each area from patient services through to scheduling to perform their expert roles more efficiently by removing duplication and rework;
3. Implementing a person-centred collaborative, inclusive, and participatory team approach to review and input into the system.

1. Collaborative Working:

The synergic principles of LSS and person-centredness, including respect for persons, gathering and listening to the VOC, facilitating staff empowerment, and observing/understanding practices were key features to the success of the project [26]. Black [2009] notes that hospitals have the added layer of being “complex social organisms”, with historical layers of power and hierarchy [32]. These social and behavioural aspects have made change management and, indeed, LSS implementation more challenging in hospital settings [33] This background makes staff engagement crucial to LSS deployment in addition to any change initiative; ultimately, they are the people who will sustain any improvement [31]. 

2. Stakeholder Engagement

Stakeholder engagement was key to the success of this project. There had been multiple previous attempts at improving both the booking form and scheduling process, with limited success. These unilateral/siloed approaches had resulted in legacy issues, such as multiple versions of the same form, processes that encouraged the duplication of work—for example, moving information from MEDITECH patient records, to paper forms, to an online scheduling app, and back to MEDITECH. LSS has been demonstrated as being effective in bringing teams together to overcome silos [16,26,34,35] and in this process improvement our approach to stakeholder engagement ensured that the existing process was scrutinised from all perspectives and that the impact of suggested improvements was considered for all participants—consultants, patient services, and scheduling teams. Whilst patients were a beneficiary, as they received earlier scheduling for surgery, they had no direct impact on the process. Each team member gained an understanding of others’ requirements in order to complete the process. There was also a new understanding that each of these requirements was equally important [32].

3. Person-Centredness

Ballé and Regnier [2007] feel that staff empowerment and culture within an organisation which encourage improvement are the cornerstones of Lean healthcare [29]. According to Drotz and Poksinska [2014], the core job characteristics for staff in a Lean environment are skill variety, increased task identity, the use of feedback, decentralised decision making, responsible autonomy, and work facilitation, where barriers to flow are removed [30]. By committing fully to appropriate training and the use of Lean, these changes can lead to positive effects for staff with respect to their working environment, individual development, and overall performance. The cross-professional nature of this teamwork decreases “hierarchical structure and boundaries between professional groups”, and employees appreciate the increased responsibilities and autonomy [30].

We also ensured that the improvements suggested had the support of a critical mass of people/key stakeholders, supporting the potential for long-term success of the new processes. If challenges are encountered there is a formal forum to discuss these, ensuring that impact solutions are considered from all stakeholders’ perspectives—preventing us from slipping to the siloed approach that had existed previously. We also have a forum for stakeholders to monitor compliance and address any issues as they arise.

We led this project utilising both adaptive and technical approaches [36]. We facilitated fast-paced technical solutions as outlined in “quick wins”, such as changing the format of the booking form and implementing simple IT solutions. We also challenged the project team to consider where an adaptive approach was required. Through stakeholder engagement and data collection, we identified where adaptive leadership and response were required to ensure the sustained success of the project—for example, the introduction of a control system for the booking form and process.

The motivation for this project was two-fold:

1. For service users: to process surgery scheduling as efficiently as possible in order to provide the correct treatment plan;
2. For service providers: create a Lean, user-friendly effective process, minimising rework with a design informed by the service provider’s needs.

Simon and Canceri [2013] described using an LSS approach to improving the process for scheduling elective orthopaedic surgeries [13]. They described many similar challenges to those we experienced, including inaccuracies in the booking form, duplication in work, and a timelapse between consultants seeing the patient and surgery scheduling. Their interventions were similar to ours in aiming for a standardised, smarter, online system to reduce inaccuracies. Similar to our results, they achieved a reduction in errors on the booking form to zero. The project team achieved the target set out at the outset. However, a limitation that must be noted is that the tight project scope to elective orthopaedic surgery narrowed the impact of the project to a very specific specialty. The success of this project has, however, resulted in hospital-wide agreement that the new process should be rolled out to all surgical bookings, using the successful combination of LSS and a person-centred approach. The project was also limited in access to IT support. Many of the solutions required IT support. The project team recommends that IT be included as stakeholders from the outset in future LSS projects.

## 5. Conclusions

Applying LSS to the process of scheduling patients for elective surgery resulted in improved time to complete surgery scheduling, reduced rework for team members, and a redesign of an improved process, serving the needs of stakeholders at each step of the process. The application of LSS allowed us to truly quantify the existing process, identifying bottlenecks, rework, and waste, and therefore produce better-informed solutions. The use of LSS methodology also had an impact on how we worked as an organisation and as a team. This project has resulted in long-term outputs for the organisation—an improved system for scheduling patients for orthopaedic surgery—which is being rolled out across other disciplines in the hospital. In addition, a shift in culture, a more open approach to the need to “understand the pain” before suggesting solutions, and a wider acceptance of the role of the VOC in understanding problems as well as designing and implementing solutions has been achieved. The success of this project has further strengthened the commitment of management to deploy LSS within the hospital, and led to more staff self-selecting for LSS training with our partner university, as the value added by the methodology is evidenced in healthcare practices. The successful use of LSS in this project will create opportunities for LSS to be utilised in other improvement processes within the hospital, but also within other healthcare institutions, which may benefit from this approach.

## Figures and Tables

**Table 1:** LSS tools.

Improvement Tool	Description of Tool	Rationale for Use in This Project
Project charter [18]	A project charter is used to define, act on, and review challenges as well as problems	It was useful in clearly identifying the goals of the project, in terms of scope
SIPOC [4]	High-level view of the process, with SIPOC standing for suppliers, inputs, processes, outputs, and customers	Identified linkages between suppliers, customers, inputs, outputs, and processes
RACI [5]	Identifies which stakeholders were responsible and accountable throughout the DMAIC phases, and which needed to be consulted or kept informed	Ensured all stakeholders were involved and engaged throughout the process improvement
CTQ [19]	Critical to quality tree: the CTQ tool is designed to capture the key measurable characteristics of a process or service whose performance standards must be met in order to satisfy the customer	Critical to quality metrics identified—length of stay, turnaround time for completion of triage, assessment, diagnostics, and decision to admit. Data availability for each metric
VOC [20]	Voice of the customer: what the customer is looking for	Identified the needs of the customers—patient, emergency department team, and organisation
Gemba [21]	Observation/understanding of where and how the work is done	Understand the process for scheduling elective orthopaedic surgery from surgeon’s consultation to surgery scheduled
FMEA [22]	Failure mode and effect analysis is a risk analysis tool that is used to prevent an event from happening. It highlights the aspects of a process that should be targeted for improvement	Prioritises/highlights the aspects of the process that should be targeted for improvement
5 S [18]	Five steps of this methodology: sort, set in order, shine, standardise, and sustain. Used to create a clean, uncluttered environment	Agree on a minimum data set and layout for booking form
TIMWOODS [23]	Acronym of transportation, inventory, movement, waiting, overprocessing, overproduction, defects, and skills. Facilitates the identification and classification of the types of waste	Identification of waste in the process

**Table 2:** Time taken to complete an online booking. Virtual Gemba descriptive statistics (*n* = 19).

Variable	N	Mean	SE Mean	St. Dev.	Minimum	Q1	Median	Q3	Maximum
Time (HH:MM)	19	25.68	6.94	30.23	0.00	0.00	22.00	50.00	96.00

**Table 3:** Results from observing an online booking/as is process.

Observations	Description	Reason
37% (*n* = 7)	Surgeries were scheduled successfully at the first attempt	All information required to complete the booking was present
26% (*n* = 5)	Rework of booking forms/surgeries was required	Rework was required due to: 50% insurance details (essential in a private hospital) and 50% laterality
37% (*n* = 7)	Scheduling was unable to be completed at the first attempt as further information was required	It was noted that these were all abandoned within 5 min of commencing an online booking as patient services identified quickly when essential information was missing, and they were all completed successfully at the second attempt

No incidence of changes to the OR schedule due to errors or omissions in the booking form was noted (*n* = 19).

**Table 4:** FMEA.

Process Steps or Product Functions	Potential Failure Mode	Potential Effects of Failure	Severity (1–10)	Potential Cause(s) of Failure	Occurrence (1–10)	Current Controls	Detection (1–10)	Risk Priority Number (RPN)	Recommended Action
Consultant completes booking form	Incomplete booking form Illegible	Unable to process booking	8	Human error, Training, Governance Duplication	10	As referred to amend and resend no data recorded	5	400	Minimum data set completion Legibility of booking form
BCO ADMIN Complete online booking	Time Incorrect data entry Admin rather than clinical staff Not live	Delayed booking Rework or impact theatre and billing Double bookings	7	Human error, Training Governance, Duplication	10	Scheduling checks all bookings Reprocessing queue	6	420	Correct procedure code and specific clinical information to minimise rework
Bookings team Complete MEDITECH	Time Incorrect data entry Admin rather than clinical staff Not live		7	Human error	5	Patient identification policy Time out Description versus code	5	175	Online booking SMART Booking APP to identify patients correctly

**Table 5:** Lean 5 S.

5 S	Before	Target
Sort	Open-text medical history fields	Specific yes/no medical history fields to highlight high-risk patients
Set in order	Layout dependant on the chronology of when the field was added	The layout reflects the flow of completion
Shine	Landscape format Limited space for the completion of fields	Portrait format in line with the rest of medical record Increased space for completion
Standardise	Sixteen different versions Paper version did not match online	One paper version which matches online
Sustain	No formal process for reviewing booking form to match users’ needs	Monthly review of forms

**Table 6:** Status pre- and post-intervention.

	Patient Services	Scheduling Team
Pre-intervention	Receive form from consultant Check completion Fill in gaps Complete an online booking Send to scheduling	Receive form from scheduling team Re-check completion Correct an online booking Transfer to MEDITECH scheduling
Post-intervention	Receive form from consultant Check completion Fill gaps Send to scheduling	Receive form from scheduling team Input to MEDITECH scheduling

**Table 7:** Time taken to complete an online booking.

Variable	N	Mean	SE Mean	St. Dev	Minimum	Q1	Median	Q3	Maximum
Time (HH:MM)	30	16.775	0.324	1.775	14.440	15.430	16.270	17.383	20.580

## Abbreviations

LSS	Lean Six Sigma
NVA	Non-value added
MDT	Multidisciplinary team
OR	Operating room
DMAIC	Define/measure/analyse/improve/control
MDS	Minimum data set
SIPOC	Suppliers, inputs, processes, outputs, customers
RACI	Responsible, accountable, consulted, and informed
CTQ	Critical to quality
VOC	Voice of the customer
FMEA	Failure mode and effect analysis
TIMWOODS	Transportation, inventory, movement, waiting, overprocessing, overproduction, defects, and skills
EMT	Executive management team
OPD	Outpatient Department
EPR	Electronic patient record
SMART	Specific, measurable, achievable, realistic/relevant, and timed
RPN	Risk prioritisation numbers
